# Polyvalent vaccines: High-maintenance heroes

**DOI:** 10.1371/journal.ppat.1006904

**Published:** 2018-04-05

**Authors:** Barbara Schlingmann, Katelyn R. Castiglia, Christopher C. Stobart, Martin L. Moore

**Affiliations:** 1 Department of Pediatrics, Emory University, Atlanta, Georgia, United States of America; 2 Department of Biological Sciences, Butler University, Indianapolis, Indiana, United States of America; 3 Children’s Healthcare of Atlanta, Atlanta, Georgia, United States of America; University of Michigan Medical School, UNITED STATES

## Introduction

Vaccines are the most efficient tools to battle infectious diseases, with an estimated prevention of 2–3 million deaths per year [1]. Vaccine development, however, is costly and challenging, especially when the target pathogen can be subdivided into serologically distinguishable types (serotypes) that individually cause disease. Broad protection against serotypes can be achieved with either polyvalent vaccines of mixed serotype-specific immunogens or by discovery and use of a good immunogen conserved among serotypes. The latter is preferable but technically elusive. The poliovirus vaccine (containing three poliovirus serotypes) was first used as a polyvalent vaccine, beginning with the establishment of the Global Polio Eradication Initiative in 1988, reducing poliomyelitis by 99% [2]. Polyvalency has been arguably more useful than using conserved immunogens to target multiple serotypes, and polyvalency has steadily advanced despite complexity and barriers to manufacturing. Here, we review challenges and developments in polyvalent vaccines.

## Challenges in polyvalent vaccine development

**Fig 1:**
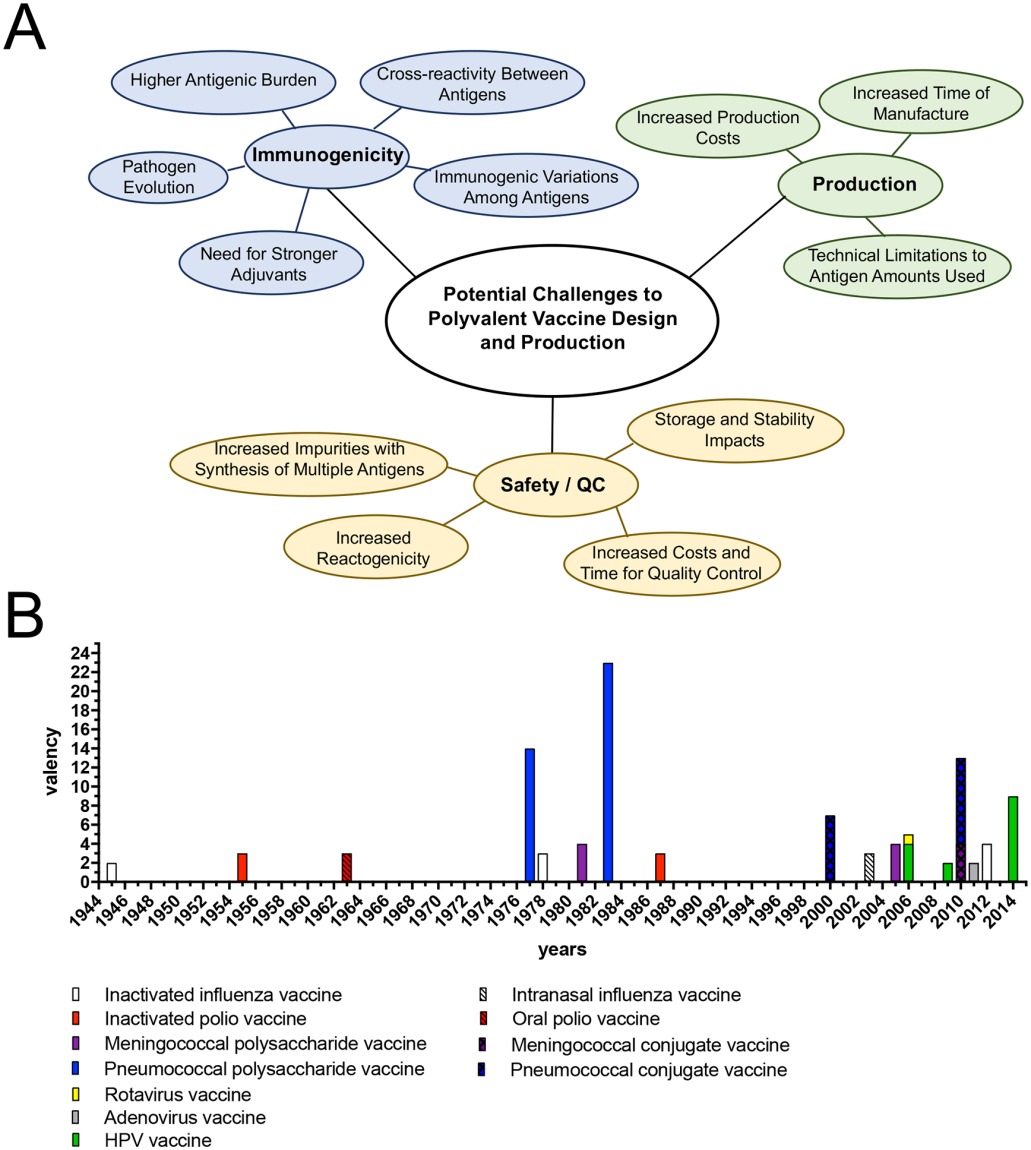
Current challenges and development of polyvalent vaccines. **(A)** Current potential challenges to development of polyvalent vaccine preparations. (**B**) Development and licensing of polyvalent vaccines in the United States. Combination vaccines are not reviewed here. Different colors indicate vaccines against the same pathogen. Blue, *Streptococcus pneumoniae* (polysaccharide vaccine and conjugate vaccine); red, poliovirus (inactivated vaccine and oral, live-attenuated vaccine); purple, *Neissaeria meningitides* (meningococcal vaccine: polysaccharide and conjugate vaccine); yellow, rotavirus (live attenuated); green, HPV, VLP; gray, adenovirus; white, influenza (inactivated and live-attenuated vaccines). Not all influenza vaccines are included. HPV, human papillomavirus; QC, quality control; VLP, viruslike particle.

Development of polyvalent vaccines poses several significant challenges (Fig 1A). During the manufacturing of polyvalent vaccines, either bulk lots of monovalent antigens are produced and then combined as a step during the manufacture, or the antigens are produced, stored separately, and mixed before administration. In either case, the individual monovalent lots and finished product must undergo quality testing measures. Compared to monovalent vaccines, the manufacture of polyvalent vaccines is associated with more exhaustive quality control measures, requiring increased investment in resources and facilities that ultimately increase costs and reduce production capacities [3]. Furthermore, manufacturers must demonstrate that there are no differences in physical, chemical, and immunological responses for simultaneous administration of multiple antigens in comparison to the individual antigens separately [4]. Live-attenuated polyvalent preparations, such as the oral poliovirus vaccine (OPV), in which the individual strains comprising the vaccine have been combined in an unequivalent ratio, must be designed to balance infectivity and immunogenicity. Combining antigens, adjuvants, and preservatives that are compatible during storage and use of the vaccine is also essential [4]. For example, preservatives used for one antigen may alter the potency (i.e., the capability of each antigen to induce neutralizing antibodies) of other antigens, buffers that are used for the different antigens may be incompatible, or titers of antibodies in patients may be lower when polyvalent vaccines are administered compared to the equivalent monovalent versions in separate administrations [3, 5]. In addition to the hurdles described, development of combination vaccines, such as diptheria, tetanus, pertussis (DTP) vaccine and measles, mumps, rubella (MMR) vaccine, which differ from polyvalent vaccines by combining antigens of different pathogens rather than serologically distinct antigens of the same pathogen, often pose additional challenges. For example, there is decreased reactogenicity associated with acellular versus cellular preparations of *Bordetella pertussis* antigen in the DTP [6].

## Balancing act for live-attenuated polyvalent vaccines: Dengue and influenza

Development of a polyvalent vaccine for dengue, a flaviviral infection caused by four dengue virus serotypes (DENV1-4), has been hindered by antibody-dependent enhancement (ADE) of disease following a heterologous secondary infection [7]. Infection with a single DENV serotype confers lasting immunity by inducing neutralizing antibodies. However, induction of suboptimal levels of neutralizing antibodies following dengue vaccine administration poses the risk of enhanced disease during natural infection [8]. The tetravalent live-attenuated DENV vaccine, produced by Sanofi-Pasteur, showed differential immunogenicity and efficacy among the serotypes and may be associated with enhanced disease in some subjects, which may be related to imbalanced infectivity [9, 10]. Similarly, the Centers for Disease Control and Prevention (CDC) Advisory Committee on Immunization Practices (ACIP) decided to not recommend administration of live-attenuated influenza vaccines (LAIVs) due to reduced efficacy and effectiveness of the new quadrivalent LAIV compared to inactivated influenza vaccine preparations [11]. The challenges to developing DENV and influenza virus vaccines exemplify how balancing compatibility and quality of the antigens in polyvalent vaccines is of special importance.

## High valency: Pneumococcal vaccines

There are currently three US licensed vaccines of 6+ valency (Fig 1): two pneumococcal vaccines (a 23-valent polysaccharide vaccine and a 13-valent conjugated polysaccharide vaccine) and the 9-valent vaccine against human papillomavirus (HPV). Pneumococcus accounts for up to 36% of all community-acquired pneumonia infections in adults but also can cause invasive diseases like bacteremia and meningitis [2]. Over 90 different serotypes of *Streptococcus pneumoniae* have been identified, based on the differences in composition of the polysaccharide capsule [2]. The first vaccine, developed in 1977, consisted of 14 different types of capsular polysaccharide (50 μg each) isolated from individually grown *S*. *pneumoniae* strains, which cause an estimated 70%–80% of cases of bacteremia and meningitis [12, 13]. Nonetheless, consideration was given to whether to include other serotypes [13]. Perceived technical difficulties to increase the valency included use of more antigenic protein load and the potential addition of impurities resulting from purification of the separate production lots of each antigen, either of which might increase adverse reactions. In 1983, Merck replaced the existing 14-valent vaccine with a new vaccine, the 23-valent polysaccharide vaccine (PPSV23) (with 90% coverage of bacteremia-causing serotypes) containing only 25 μg of each antigen per dose [2]. Clinical trials had shown similar immunogenicity and no negative immunogenic effects by the introduction of additional serotypes [2, 13].

In children (≤2 years) and immunocompromised patients, the pneumococcal polysaccharide vaccine did not consistently generate immunity [2]. In 2000, a 7-valent conjugate vaccine was licensed (PCV-7). The introduction of the 7-valent pneumococcal vaccine (types 4, 6B, 9V, 14, 18C, 19F, and 23F.) significantly reduced invasive pneumococcal disease caused by these serotypes [14]. However, it led to the emergence of the noninvasive serotype 19A that had been associated with antibiotic resistance [14, 15]. In 2010, Pfizer introduced PCV-13 (Prevnar), which contains six additional antigens (from types 1, 3, 5, 6A, 19A, and 7F) [2]. The conjugate vaccine is composed of the virulent *S*. *pneumoniae* serotype-specific polysaccharides that are chemically linked to a nontoxic recombinant version of the diphtheria toxin (carrier protein CRM_197_) that is used to enhance the immune reaction in children. PCV-13 contains serotypes responsible for only 61% of all invasive pneumococcal disease cases in children younger than 5 years [2], suggesting a need for a further increase in valency. However, there remained concerns with development of a conjugate vaccine with higher valencies, because elevated carrier protein levels could impair the antibody response through antigen competition or epitope suppression [16, 17]. Furthermore, the manufacturing process requires costly containment facilities, and the chemical conjugation can result in a heterogeneous product, which increases cost of quality control. Development efforts toward the use of pneumococcal proteins as antigens instead of polysaccharides [17], or novel conjugation technologies such as bacterial N-linked protein glycosylation to produce glycoconjugate antigens [18], may be a strategy that could overcome problems with current pneumococcal polyvalent marketed vaccines.

## Increasing valency: HPV vaccines

HPVs are the main cause of cervical cancer. Over 150 HPV serotypes have been identified based on sequence differences of the outer capsid protein L1. Forty HPV types are capable of infecting mucosal epithelium and are grouped into non-oncogenic and oncogenic serotypes according to their epidemiologic association with cervical cancer [2]. In the US, there are three HPV vaccines licensed: Gardasil (2006), a quadrivalent vaccine against HPV types 6, 11, 16, and 18 [19]; Cervarix (2009, discontinued in 2016), a bivalent vaccine against HPV types 16 and 18 [20]; and Gardasil 9 (2014), a 9-valent vaccine against HPV types 6, 11, 16, 18, 31, 33, 45, 52, and 58 [21]. The vaccines consist of recombinant L1 proteins that self-assemble into highly immunogenic viruslike particles (VLPs). The L1 protein VLPs are either produced by separate fermentation of recombinant yeast (*Saccharomyces cerevisiae*) or by expression in an insect producer cell line infected with baculovirus containing the L1 gene [20, 21]. Cervarix contains a proprietary adjuvant, AS04, while Gardasil is adjuvanted with aluminum hydroxyphosphate sulfate [20, 21]. Both Gardasil and Cervarix confer immunity against HPVs 16 and 18, which are responsible for 70% of all cervical cancers, while Gardasil also immunizes against two HPV types that cause about 90% of genital warts [2]. With the addition of the five HPV strains in Gardasil 9, the vaccine now provides protection against HPV serotypes responsible for up to 90% of cervical cancers [2]. Clinical studies showed that the efficacy for Gardasil 9 was similar to the 4-valent vaccine; however, the frequency of adverse events was higher, likely due to higher amounts of HPV VLPs and adjuvant [22]. It remains to be determined if this potentially limits a further increase of valency for HPV vaccines.

It was unclear whether Gardasil 9 would benefit previously HPV-vaccinated individuals. Preliminary results showed that antibody titers for the five added serotypes were lower in individuals who had previously received Gardasil, suggesting potentially reduced protection against the additional serotypes for such individuals, although effective titers were reached [23]. A future direction for the development of next generation HPV vaccines may include the use of fewer, but more cross-protective, antigens, such as the well-conserved L2 protein of the virus. The L2 protein alone was shown to have low immunogenicity compared to the VLPs; however, combining L2 epitopes with L1 VLPs may be an approach to achieve broad protection against HPV [24, 25].

## Experimental 50-valent rhinovirus vaccine

Rhinoviruses (RVs) are the predominant cause of respiratory tract illnesses globally and are thought to cause roughly one quarter of asthma and chronic obstructive pulmonary disease (COPD) exacerbations [26]. There are three RV species (A, B, C) with 80 A, 32 B, and 55 C serotypes [27]. Co-circulation of many of these RV types and the fact that past studies showed little cross-reactivity between the types (immunity is serotype specific) discouraged vaccine development for decades [28]. Efforts to create a subunit vaccine with recombinant RV capsid protein or shorter peptides (VP1 and VP3) showed that cross-reactive neutralizing antibodies can be induced in rabbits. However, the antibodies isolated from the rabbits immunized with RV-14 derived peptides (VP1 and VP3) neutralized only 25%–50% [29, 30] of other serotypes tested, making it unlikely to achieve effective protection against all RV species with a single antigen [28]. Although trials with only one type of formalin-inactivated RV demonstrated sufficient protection [31], RV vaccine development was inhibited by the large number of RV serotypes that would be needed. However, recently, animal studies of a 25-valent RV vaccine in mice and a 50-valent vaccine in rhesus macaques, both adjuvanted with alhydrogel, showed that immunogenicity against each RV serotype correlated with the antigen amount for each serotype used in the vaccine [32]. The valency of the vaccine itself did not decrease immunogenicity of the vaccine, showing that a polyvalent RV vaccine is immunologically possible.

## Conclusions

Polyvalency is a challenging but often necessary vaccine approach, and its success is a function of cost of goods, technical feasibility, and consistent efficacy. Strategies to overcome these challenges, such as the use of antigens that have the ability to induce immunity across different serotypes, are currently being employed [17, 24, 25, 29, 30]. Current investigations into broadly neutralizing antibodies have provided new focus on potential conserved epitopes. Yet, the induction of serotype cross-reactivity remains limited, and recombinant, epitope-focused approaches are not necessarily faster or more fruitful [29, 30]. So far, there are only a handful of recombinant protein vaccine antigens, such as hepatitis B. There are 82 vaccine products licensed in the US. Of these, 35 are polyvalent (Fig 1B). The frequency and overall valency associated with vaccine development appear to be increasing. Collectively, the need for potent and broadly efficacious vaccine preparations highlights the importance of this yeoman’s work in vaccine development.
